# Early and Late Complications Related to Central Venous Catheters in Hematological Malignancies: a Retrospective Analysis of 1102 Patients

**DOI:** 10.4084/MJHID.2014.011

**Published:** 2014-02-14

**Authors:** Salvatore Giacomo Morano, Lorenzo Coppola, Roberto Latagliata, Paola Berneschi, Antonio Chistolini, Alessandra Micozzi, Corrado Girmenia, Massimo Breccia, Gregorio Brunetti, Fulvio Massaro, Giovanni Rosa, Pietro Guerrisi, Franco Mandelli, Roberto Foà, Giuliana Alimena

**Affiliations:** 1 Dipartimento di Ematologia, Oncologia, Anatomia Patologica e Medicina Rigenerativa, Azienda Policlinico Umberto I, “Sapienza” Università di Roma, Italy.; 2 Istituto di Ematologia “AOU” Sassari, Italy.; 3 Dipartimento Anestesia e Rianimazione, Azienda Policlinico Umberto I, “Sapienza” Università di Roma; 4 Dipartimento Scienze Radiologiche, Azienda Policlinico Umberto I, “Sapienza” Università di Roma

## Abstract

Several severe complications may be associated with the use of central venous catheters (CVC). We retrospectively evaluated on a large cohort of patients the incidence of CVC-related early and late complications. From 7/99 to 12/2005, 1102 CVC have been implanted at our Institution in 881 patients with hematological malignancies (142,202 total day number of implanted CVC). Early mechanic complications were 79 (7.2% - 0.55/1,000 days/CVC). Thirty-nine episodes of early infective complications (<1 week from CVC implant) occurred (3.5% - 0.3/1000 days/CVC): furthermore, 187 episodes of CVC-related sepsis (17% - 1.3/1000 days/CVC) were recorded. There were 29 episodes (2.6%) of symptomatic CVC-related thrombotic complications, with a median interval from CVC implant of 60 days (range 7 – 395). The rate of CVC withdrawal due to CVC-related complications was 26%. The incidence of CVC-related complications in our series is in the range reported in the literature notwithstanding cytopenia often coexisting in hematological patients.

## Introduction

Central venous catheters (CVC) are commonly employed in the management of hematological malignancies. Their use is crucial in order to have a safe and easy venous access, for blood sampling, drug infusions, supportive iv treatments, blood product administration and parenteral nutrition all along the course of the disease; in addition, CVC are helpful in some therapeutic procedures, such as stem cell collection and apheresis.

However, the use of CVC is often associated to several complications, that can lead to a device malfunctioning and/or to an increased patient morbidity with longer hospital admissions and more expensive medical assistance.^1^,^2^

CVC-related complications can be divided into early complications (mechanical and infective) and late complications (mechanical, infective and thrombotic); early complications are generally secondary to the insertion procedures, while late complications are more frequently due to malpractice in the CVC management during the follow-up.

Several studies have addressed the incidence of infective and thrombotic CVC-related complications in patients with solid tumors; on the contrary, at present only few data are available in hematological malignancies, even if patients in this setting are quite different from patients with solid tumors due to the occurrence of more severe and prolonged cytopenia.^3^,^4^

The aim of this retrospective study was to evaluate the rate of early and late CVC-related complications in a large cohort of patients with acute and chronic hematological malignancies, followed at a single Institution with a specific team dedicated to CVC insertion and management.

## Patients and Methods

### Patient Population

All consecutive patients with acute and chronic hematological malignancy followed at our Institution, who received a CVC insertion between July 1999, and December 2005 were collected in the present retrospective analysis.

Prior to CVC insertion, all patients were screened with a complete blood count, coagulation tests and chest radiography to rule out conditions contraindicating a CVC insertion. Patients with a platelet count <50 × 10^9^/l received platelet infusion before the insertion.

### CVC Characteristics and Insertion

A single type of tunneled CVC (Groshong-Bard, monolumen 7 Fr) was used. This long-term, silicone, valved CVC was preferred to polyurethane, non-valved CVCs in consideration of a presumed reduced thrombotic risk as compared to polyurethane and non-valved devices. All insertion procedures were performed in a surgical ward under strict aseptic conditions and maximal barrier precautions: no sterile cap, no sterile facemask, sterile gloves, sterile body gowns, careful skin antisepsis and vast draping of the procedure area. Povidone-iodine (10% iodine) was used as a skin antiseptic for all the procedures; two preliminary washings were performed, and the solution was kept on the skin for two minutes in each one. All insertion procedures were performed by the same clinician with the assistance of a professional nurse, using a percutaneous route according to the Seldinger-peel-away technique. Local anesthesia or light sedation was used during the insertion, and the distal CVC portion was positioned at the atrio-caval junction under brilliance amplification guide.

The day after the insertion, 2nd chest radiography was performed to confirm the correct position of the distal CVC tip and to exclude early complications.

The CVC management and the weekly medications were performed by the ward nurses when the patients were hospitalized and by the same dedicated team which inserted the CVC in case of patients discharge.

For each patient, a chart was provided by a dedicated team in which all data regarding CVC management and related complications were timely recorded.

### Definition of CVC-related Complications

The CVC-related complications were evaluated as early complications if they occurred in the first week since the CVC insertion; all complications occurring thereafter were considered as late complications.

The following types of events were considered as mechanical complications: impaired or unsuccessful venipuncture, pneumothorax, arterial puncture, hematoma, dislocation, obstruction, pinch-off, malfunction (defined as persistent pain on infusion and/or inability to infuse or withdraw after an initial period of a correct CVC function), accidental removal, and breakage/leakage.

A diagnosis of deep venous thrombosis (DVT) required the direct visualization of the thrombus at the ultrasound or CT-scan examination with lack of compressibility by probe pressure and/or absence of spontaneous flow by Doppler and/or absence of phase flow with respiration. A diagnosis of superficial thrombophlebitis was made when at least one of the following signs was evident: local induration or erythema, warmth, pain or tenderness along the CVC vein.

The criteria according to the guidelines of the Centers for Disease Control were employed to define infective CVC-related complications,. These criteria required evidence of fever and/or local symptoms (hyperemia, local pain) associated with positivity of microbiological cultures (blood cultures, semi-quantitative cultures of the tip) when the same organism was isolated from the CVC tip and the peripheral blood sample, with no apparent source of infection other than CVC.

The unit of analysis was the central line within a patient, and CVC related complication rate was defined as the number of adverse events divided by number of CVCs and the number of central line-days during the period from CVC insertion to the onset of the adverse event, multiplied by 1000.

## Results

### Patient Population

Between July 1999 and December 2005, 1,102 CVC have been inserted in 881 patients (503 males and 378 females, median age 44.4 years, range 1 – 87 years) with hematological malignancies followed at our Hematological Department and treated with chemotherapy and/or stem cell transplantation. The distribution of the different hematological diseases is shown in Table 1. The CVC was inserted more than once during the course of the disease in 176 patients (range 2–5 insertions).

The CVC insertion was performed in the right subclavian vein, in 883 cases (80.1%), and in the left subclavian vein, in the remaining 219 cases (19.9%).

Mean duration of catheterization was 131.76 days (145,202 CVC-days), range 5-1.651. The CVC was removed due to the completion of the treatment plan in 706 (64%) of cases and due to death in 110 (10%) of cases; CVC-related complications led to the CVC withdrawal in the remaining 286 (26%) of cases.

### Early CVC-related Complications

There were 79 episodes (7.2% - 0.54/1000 catheter days) of early mechanical complications; the different types of mechanical complications and their relative frequencies are reported in Table 2. Among the 32 episodes of impaired venipuncture, an echo-Doppler examination revealed stenosis or thrombotic obstruction of the vessel in 14 cases.

There were 39 episodes (3.5% - 0.3/1000 catheter days) of early infective complications. A different distribution of such complications according to the year of CVC insertion was reported, ranging from 9 episodes in 1999 to only 1 episode in 2004; moreover, no episode was reported in the first 9 months of 2005.

In these cases, the underlying hematological malignancy was AML in 31 patients, ALL in 3 patients, NHL in 3 patients and MM in 2 patients. In 32/39 episodes (82.0%), the absolute number of polymorphonuclear cells (PMN) was lower than 0.5 × 10^9^/l. The incidence of different infective agents is reported in the Table 3.

### Late CVC-related Complications

Among the late mechanical complications, there were 101 episodes (9.1%) of CVC malfunctioning, due to clots and/or aggregates obstruction (43 cases, of which 34 resolved with urokinase), fibrin-sheath (24 cases) and pinch-off (3 cases); no apparent cause was identified in the remaining 31 cases. Moreover, there were 24 episodes (2.1%) of accidental removal, 11 episodes (0.9%) of dislocation and 5 episodes (0.4%) of extra-vessel rupture.

Apart from episodes of clot obstruction and fibrin-sheath already reported among mechanical complications, there were 29 episodes (2.6%) of symptomatic DVT (23 in the right and 6 in the left subclavian vein). The underlying hematological malignancy was AML in 10 patients, NHL in 9 patients, ALL in 3 patients, CLL, HD and MM in 2 patients, respectively, and CML in 1 patient.

The median interval between the CVC insertion and the onset of thrombotic complication was 60 days (range 7 – 395 days). Six of the 29 patients who developed thrombotic complications had a previous CVC insertion. When the thrombotic episode occurred, the platelet count was <50 × 10^9^/l in 9/29 patients. A complete thrombophilia screening was performed in all 29 patients with CVC-related thrombosis and always proved negative.

Low-weight molecular heparins were employed in all patients with CVC-related thrombosis and CVC extraction was performed only after echo-Doppler evidence of vessel recanalization. The thrombotic episode was never complicated by pulmonary embolism.

With regard to late infective complications, 187 episodes (17% - 1.3/1000 catheter days) were recorded; among these episodes, 138 (73%) were CVC-related sepsis which always needed a CVC extraction, while 49 (27%) were infections of the subcutaneous tunnel and/or exit-site, which required a CVC extraction in 5 cases only. When the late infective complication occurred, the PMN count was <0.5 × 10^9^/l in 90 patients (48.1%), between 0.5 and 1.0 × 10^9^/l in 39 patients (20.8%) and >1.0 × 10^9^/l in the remaining 58 patients (30.1%). The incidence of different infective agents is reported in the Table 3.

## Discussion

Management of onco-hematological patients has profoundly changed following the introduction in the clinical practice of CVC, which has enabled an easier planning of chemotherapy, transplant procedures, blood sampling and intravenous supportive care. However, their routine use is also linked to a number of CVC-related complications. Among the complications commonly reported in literature on Groshong CVCs, infections are the most frequent (0.1–11.5 per 1000 CVC days),^5^,^6^ although mechanical problems, including thromboembolic accidents, may occur at a non-negligible rate (1.2–13%).^7^,^8^ However, these data primarily refer to adult patient populations with solid tumours.

In the present study, we retrospectively evaluated the incidence of such complications among a large consecutive series of patients with hematological diseases, taking into account the peculiar setting in which all CVC were implanted. The most important aspect of our study is represented by the uniformity in the management all along the CVC life history, i.e. the same type of CVC implanted, in the same vein by the same team with the same technique; moreover, post-implantation management was carried out by the same dedicated team, using international standardized guidelines.^9^

As a matter of fact, uniformity is crucial in early mechanical complications, the occurrence of which is due to differences in many factors like medical expertise, type of device and type of technique or vessel employed. In our study, this uniformity translated into a rather low global incidence of mechanical complications (7.2%), with a very low rate of pneumothorax (0.27%) that is in agreement with literature data.^10^

Late mechanical complications occurred overall in 141 out of 1,102 CVCs (12.8%); they included only 101 malfunctions due to occlusion (partial or complete), 5 episodes of CVC external tract rupture, 24 accidental removals, 11 dislocations. Results reported in the literature show that the Broviac/Hickman device develops mechanical complications with a variable incidence of 1.3–10.1 episodes/1,000 CVC day (3,6–77,6% of CVC).^7^,^14^

Thrombotic complications have been evaluated in many clinical studies. However, these studies are very heterogeneous regard to clinical endpoints, follow-up and definition of CVC-related symptomatic and/or asymptomatic thrombosis. Thus, the real rate of incidence is still undefined since based on the different diagnostic approaches utilized.

In our study, an ultrasound examination was required to confirm a CVC-related thrombosis; with such a definition, the rate was 2.6%, that is in agreement with literature data (range 1.2 – 13%).^11^ It is worth noting that no patient with CVC-related thrombosis was positive at the thrombophilia screening. The role of a low platelet level in preventing CVC-related thrombosis is still unclear: in our experience, 9 of the 29 patients had platelet levels <50 × 10^9^/l when the thrombosis occurred, thus outlining that thrombocytopenia reduces but does not eliminate a thrombotic risk, as previously reported.^3^

The incidence of early infective complications was 3.5%. There was, however, a progressive decrease during the study period, from 9 episodes in 2000 to no episode in 2005; this finding is probably due to better practical expertise of the team during the time framework of the study.

On the whole, we observed 187 late infective episodes (17% - 1.3/1000 catheter days); 76% of these episodes were observed in patients with PMN <1.0 × 10^9^/l. Our data are comparable with those from the literature, where the incidence of late infective complications in the hematological setting ranges from 0 to 27.7%, according to the different features of patients and devices^11^–^15^ and is higher in patients with neutropenia, CVC-related thrombosis and during transplantation procedures.^13^,^14^

## Conclusions

Our data emphasize the safety and efficacy of Groshong CVC all along the treatment course in patients with hematological diseases. Furthermore, the use of a dedicated team with a progressively increasing expertise was capable of reducing the rate of early and late CVC-related complications, also in this subset of patients commonly affected by cytopenia; this seems to translate into a long period of CVC employment that in our experience covered all treatment programs in more than 75% of patients.

A clear limit of the present study is represented by the retrospective analysis although all consecutive patients who received CVC implantation in our Institute were considered and by the difficult comparison with other reported experiences. A prospective survey with specific evaluation of risk factors is thus warranted.

## Figures and Tables

**Table 1:** Hematological diseases.

	N∘	%
Acute myeloid leukemia	359	40.7%
Non-Hodgkin lymphoma	225	25.5%
Acute lymphoid leukemia	90	10.2%
Multiple myeloma	88	10%
Hodgkin lymphoma	56	6.35%
Chronic myeloid leukemia	26	3%
Chronic lymphocytic leukemia	21	2.4%
Other malignancies	16	1.8%

**Table 2:** Early mechanical complications.

Type of complication	N∘ (%)	(catheter days)
Impaired venipuncture	32 (2.9%)	0.22/1000
Arterial puncture	16 (1.4%)	0.11/1000
Pneumotorax	3 (0.27%)	0.02/1000
Air embolism	1 (0.09%)	0.006/1000
Hematoma	27 (2.4%)	0.18/1000

**Table 3:** Infective complications: microbial agents in early and late events.

Microbial agent	Relative rate of incidence, n (%)	
	Early infections (39 episodes)	Late infections (187 episodes)
Coagulase-negative Staphylococci	20 (51.3)	56 (29.9)
Pseudomonas species	10 (25.6)	47 (25.2)
Sternotrophomonas maltophylia	6 (15.4)	30 (16.1)
Escherichia coli	/	15 (8.0)
Staphylococcus aureus	3 (7.7)	14 (7.5)
Enterococcus faecalis	/	9 (4.8)
Candida species	/	8 (4.3)
Enterococcus	/	4 (2.1)
Klebsiella pneumoniae	/	4 (2.1)
